# An Improved Space-Based ISAR Simulation Method Using Two-Line Element Data

**DOI:** 10.3390/s26144338

**Published:** 2026-07-08

**Authors:** Wenjie Zhu, Hongxing Hao, Ronghuan Yu, Desheng Liu

**Affiliations:** National Key Laboratory of Space Target Awareness, Space Engineering University, No.1 Bayi Road, Huairou, Beijing 101400, China; momoiairi520@hgd.edu.cn (W.Z.);

**Keywords:** alternating direction method of multipliers (ADMM), inverse synthetic aperture radar, radar simulation

## Abstract

Inverse synthetic aperture radar (ISAR) technology is widely used in the field of target recognition, and radar simulation technology is also being extensively studied. The present study focuses on the digital simulation of ISAR technology and proposes a sparse imaging simulation method for spatial targets based on two-line element from satellites. The method utilizes two-line element (TLE) data from satellites as a foundation and applies an improved alternating direction method of multipliers (ADMM) for echo data processing. It enables high-resolution imaging in a simulation environment while accurately resolving the motion state of space targets relative to the radar line of sight. The present study analyzes data such as image entropy, image contrast, and normalized root mean square error for the imaging results. The proposed simulation method offers the advantages of high-sparsity imaging and low signal-to-noise ratio (SNR) imaging, enabling better simulation and application of inverse synthetic aperture radar.

## 1. Introduction

With the development and advancement of computer technology, digital radar simulation has been widely studied due to its experimental reproducibility [1]. In the field of space target inverse synthetic aperture radar research, acquiring images through digital simulation and radar modeling overcomes the limitations of space field conditions compared to obtaining space target images via actual radar equipment. It enables the generation of radar signal images under extreme conditions that are difficult to produce on actual equipment, saving both time and resources. The imaging source is flexible and controllable, offering a high cost-effectiveness ratio, while also allowing for continuous repetition of experiments, which facilitates the validation of imaging algorithms. Radar detection technologies and architectures are continuously evolving, and sparse image reconstruction techniques for inverse synthetic aperture radar based on compressed sensing theory are being widely applied [2]. With the advancement of compressed sensing theory, as well as progress in solution algorithms and research on regularization methods [3]. This imaging algorithm, based on compressed sensing theory, is currently undergoing extensive validation and development using digital radar simulation technology. While sparse coding algorithms are advancing rapidly, there is an increasingly urgent need for sparse signal recovery in the field of radar simulation and development. This raises the necessity of validating high-performance imaging metrics—such as detection performance and anti-interference capabilities—of sparse coding technology in radar signal processing [4]. This necessitates that images generated by sensors such as radar and optoelectronic systems possess a very high degree of realism and practicality. Consequently, radar image simulation for modeling has attracted widespread attention from scholars and extensive research has been conducted in this area. Furthermore, with the development of new-generation radar systems, frequency-domain sparse radar and dual-base radar have emerged. As noted in Reference [5], during the current development of new-generation radar systems for space targets, certain characteristics of radar transmit/receive branches and the need to switch beams when observing targets can result in opportunities for echo recovery, enabling the formation of sparse apertures. Therefore, in the development of radar applications for space targets, it is necessary to propose a reasonable and effective digital twin radar simulator.

Radar simulators, as a product of the integration of simulation technology and radar technology, represent an important branch of systematic scientific simulation applications. Simulating radar signal processing and data processing workflows allows for a realistic depiction of radar operating states and processes and enables cost-effective alternatives to real-world imaging of space targets [6]. Inverse synthetic aperture radar (ISAR) simulation systems have made significant strides and have played a crucial role in algorithm research and equipment development [7]. Examples include the multi-mode radar simulators developed by Presagis and Boeing, who have also made significant strides in SAR radar simulation [8]; for example, Boeing’s multi-mode radar simulator is capable of simulating ISAR. These simulation systems play a critical role in training and research and development.

The core of digital simulation lies in simulation algorithms. Current research aims to improve radar efficiency and robustness and therefore focuses on sparse imaging algorithms. Sparse scenarios typically arise from factors such as radar transmission frequencies and complex electromagnetic frequencies. As noted in Reference [9], the distributed transmission and reception characteristics of dual-base systems, combined with the need to continuously switch beams when observing targets, can easily lead to missing echoes during the observation process, thereby resulting in sparse apertures. To solve such problems, numerous algorithms have been developed; among them, the ROMP algorithm [10] was proposed for acquiring high-resolution inverse synthetic aperture radar (ISAR) images, while Reference [11] proposed an algorithm using Bayesian dual-base ISAR to acquire images under low signal-to-noise ratio conditions. Zhang S et al. [12] proposed a sparse ISAR self-focusing imaging algorithm based on sparse Bayesian learning, while Reference [13] proposed the use of the 2D-ADMM algorithm to solve constrained optimization problems and achieve target image reconstruction.

This work addresses the current state of ISAR simulation research both domestically and internationally, drawing inspiration from deep-space telemetry, tracking, and command data from various satellite systems [14]. Using the ISAR simulation imaging method in 3D space [15,16] to establish a spatial scatterer model, abstract 3D scatterer information is directly extracted from CAD models and applied to simulation imaging using compressed sensing theory [17]. To address the sparse image reconstruction problem for inverse synthetic aperture radar (ISAR) of space targets, this paper first establishes a radar echo simulation method based on TLE data, followed by sparse sampling and the application of an appropriate spatial noise model. Subsequently, a sparse image reconstruction algorithm based on an improved Alternating Direction Method of Multipliers (ADMM) is proposed. Finally, the imaging results of the proposed method and various imaging algorithms are analyzed under different noise levels and sparsity conditions, and the imaging results of space targets are examined across different observation times. This validates the unique advantages and high-performance simulation capabilities of space target imaging simulation based on TLE data and the improved alternating direction method of multipliers (ADMM).

## 2. Simulation of Radar Echo Data Based on TLE Data

TLEs are sets of average orbital parameters fitted using the SGP4 model. This includes elements such as epoch, inclination, eccentricity, and mean motion—which reflect the shape, position, and decay trend of the orbit—as well as the atmospheric drag fitting coefficients. These average roots must be calculated using the analytical solution of the SGP4 model in order to be converted into the satellite’s instantaneous position and velocity. The method employs TLE data to simulate radar echo data as follows. First, based on the TLE data of the space target and taking Earth’s occlusion into account, the radar’s line-of-sight to the target is determined. Second, during the calculation of the echo matrix, echo data is computed based on the TLE data and the accumulated number of pulses. This imaging simulation method relies on comprehensive parameters beyond the space target’s motion trajectory to estimate constraints and fully utilizes the effects of atmospheric drag perturbations, non-spherical orbit perturbations, long-period perturbations, and short-period perturbations in the TLE data on the orbit and imaging. The method is characterized by the generation of an ISAR echo matrix for the space target based on the original TLE data and the scatterer model, in combination with radar parameters, specifically including the following steps:

1. Obtain the positions of scattering points and scattering parameter information based on the three-dimensional scattering model of the space target;
2. Calculate the position and velocity of the space target using the SGP4 model based on the space target’s TLE data;
3. Calculate the echo matrix based on the positions of the space target’s scattering points.

The accuracy of the SGP4 model is highly dependent on the orbit type and extrapolation time. For low Earth orbit (LEO), positional errors are typically around 1–3 km near the ephemeris time, while extrapolation errors over a 24-h period can reach 1–5 km. However, when extrapolated beyond 3–7 days, or when used for medium-to-high orbits (GEO), the error increases significantly to tens of kilometers. This is particularly true during periods of intense solar activity or following satellite maneuvers, when TLEs rapidly become obsolete and are no longer suitable for high-precision applications. Since imaging is a process performed near the ephemeris time, the errors in key data required for imaging—such as satellite velocity—calculated using the SGP4 model are acceptable and effective. In Step 2 described above, the SGP4 analytical orbit model is applied based on the TLE data of the space target. The SGP4 analytical orbit model consists primarily of four parts:

- Calculation of the initial mean velocity and semi-major axis;
- Calculation of atmospheric drag perturbations and non-spherical orbit perturbations;
- Calculation of long-period perturbation terms;
- Calculation of short-period perturbation terms.

According to the SGP4 model, the spatial position of the target is calculated as $O_{p}\;=\;(O_{u},{\;O}_{v},{\;O}_{w})$, the linear velocity as v = (u, v, w), the orbital linear velocity as $v_{n}\;=\;{\left\|v\right\|}_{2}$, and the angular velocity as $w_{n}\;=\;v_{n}\;/\;r_{o}$, where $r_{o}\;=\;{\left\|O_{p}\right\|}_{2}$. The radar position is denoted as $R_{p\;}=\;(R_{u},{\;R}_{v},{\;R}_{w})$.

The pseudocode for calculating the echo matrix in Step 3 is shown in the table below, see Algorithm 1:
**Algorithm 1:** Steps for Generating a Simulation Echo Matrix Based on TLE Data.**Input:** $M_{\mathrm{re}}\;=\;{0\;}^{N\times M}$, N > 0$,\;M\;>\;0,\;R_{\mathrm{range}}\;=\;{0\;}_{1\times M\;}$, i = 0, j = 0 **T**_range_ = [0, 1/*f*_range_, 2/*f*_range_…, T_pulse_ − 1/*f*_range_] − T_pulse_/2, *f*_range_ > 0, T_pulse_ > 0.**Output:** $M_{\mathrm{re}}\in C^{M\times N}$ 1. **Begin** 2. **While** i < *N* 3.    i = i + 1 4.    sec =(i−N / 2)/ PRF 5.    $O_{pi}\;=\;(O_{ui},{\;O}_{vi},{\;O}_{wi})$ 6.    $v_{i}\;=\;(u_{i},\;v_{i},\;w_{i})$ 7.    $v_{ni}\;=\;{\left\|v_{i}\right\|}_{2}$$,\;r_{oi}\;=\;{\left\|O_{pi}\right\|}_{2}$ 8.    $w_{ni}\;=\;v_{ni}\;/r_{oi}$ 9.    **While** j < *M* 10.    j = j + 1 11.    $R_{s}(j)\;={\left\|P_{s}{\left(j,\;:\right)}^{T}\;+{\;O}_{\mathrm{pi}}\;–\;R_{p}\right\|}_{2}$ 12.    $R_{\mathrm{range}}\;={\;R}_{\mathrm{range}}\;+\;\exp\;(-4\pi j\;/\;c\;(f_{c}\;+\;\gamma \;(T_{\mathrm{range}}\;–\;2r_{\mathrm{oi}}\;/\;c\;))(R_{s}\left(j\right)–r_{\mathrm{oi}}))$ 13.    **end** 14.    $M_{\mathrm{re}}(i,\;:)\;={\;R}_{\mathrm{range}}$ 15. **end** 16. **end**

**M**_re_ represents the simulated ISAR echo matrix constructed based on TLE data, **R**_range_ represents the simulated range-domain echoes, and **T**_range_ represents the time slice. A sparse model is constructed using a randomization approach, where I(⋅) denotes the column selection function for the matrix. A proportion of columns equal to K is randomly selected from the matrix and reset to a zero vector to simulate the sparsity of the echo matrix.

Various methods are available for selecting noise models. This study adopts the Milton A noise model, which better accounts for the actual noise conditions in the atmosphere. The mathematical expression for this noise model is as follows, and it better reflects the actual electromagnetic noise environment in the atmosphere. Noise is defined as ε, and finally, the echo matrix S is generated, with the following mathematical expression:

(1) $$S\;=\;I(M_{\mathrm{re}})\;+\;\varepsilon_{0},$$

## 3. An Improved Alternating Direction Method of Multipliers

The target scatter point reflection signals generated from the TLE data in the previous section can be expressed as

(2) $$s({\;f}_{m},\;t_{n})=\sum_{k=1}^{K}{\;\sigma }_{k\;}\exp(-j4\pi f_{m}\;R_{k}(t_{n})/c)+\varepsilon (f_{m},{\;t}_{n}),$$

where *f*_m_ is the signal frequency, defined as *f*_m_ = *f*_c_ + (m – 1)Δ*f*, *f*_c_ is the center carrier frequency, m = −(M − 1)/2, ⋯, M/2, M is the cumulative number of pulses, and Δ*f* is the frequency-domain sampling interval. *T*_n_ is the discrete observation time, n = 1, 2, ⋯, N, where N is the number of pulses, K is the number of target scatterers, ε(*f*_m_, *t*_n_) is noise, and **R**_k_(*t*_n_) represents the distance between the kth scatterer and the radar. It can be expressed as

(3) $$R_{k}(t_{n})=R_{0}+x_{k}-y_{k}Ω{\;t}_{n},$$

where **R**_0_ is the distance between the origin of the target coordinate system and the radar, Ω is the target’s angular velocity, and (*x*_k_, *y*_k_) are the coordinates of the scatterer in the target coordinate system.

Therefore, the reflected signal from the scattering points of the observed target can be further described as follows:

(4) $$s({\;f}_{m},\;t_{n})\;=\sum_{k=1}^{K}{\;\sigma }_{k\;}\exp(-j4\pi \;f_{m}\;x_{k}/c)\exp(-j4\pi \;f_{m}y_{k}Ω{\;t}_{n}/c)+\;\varepsilon (f_{m},{\;t}_{n}),$$

The problem simplifies as following expression, where the formula I(Echo) = $F_{a}XF_{r}^{T}$:

(5) $$S\;=\;F_{a}XF_{r}^{T}\;+\;E,$$

In particular, **S** is the vector generated via column-vector prioritization for the observation matrix; **x** is the vector generated via column-vector prioritization for the scattering coefficients, which also represents the original image to be reconstructed; and **E** is the vector generated via column-vector prioritization for the noise matrix.

**F**_a_ is the directional Fourier matrix, defined as

(6) $$F_{a}=\left[\begin{array}{cccc} 1 & 1 & \cdots & 1\\ 1 & \omega & \cdots & \omega^{(P-1)}\\ \vdots & \vdots & \ddots & \vdots \\ 1 & \omega^{(N-1)} & \cdots & \omega^{(N-1)(P-1)} \end{array}\right],$$

Here ω = exp(−j2π/*N*) and *P* represents the image height.

**F**_r_ is the distance-oriented Fourier matrix, defined as

(7) $$F_{a}=\left[\begin{array}{cccc} 1 & 1 & \cdots & 1\\ 1 & v & \cdots & v^{(Q-1)}\\ \vdots & \vdots & \ddots & \vdots \\ 1 & v^{(M-1)} & \cdots & v^{(M-1)(Q-1)} \end{array}\right],$$

Here *v* = exp(−j2π/*M*) and *Q* represents the image width.

Therefore, the key problem now is to solve for **X** given that **S**, **F**_a_, and **F**_r_ are known from Equation (5). This problem can be further formulated as the following minimization problem.

(8) X^= argmin X12S – FaXFrTF2+λX1 ,

In this problem, the first term is the error term and the second term is the sparsity-inducing term; this minimization problem can be further transformed into a constrained problem.

(9) X^= argmin X12S − FaXFrTF2+λB1, s.t. X=B,

The study employs the exchange multiplier method as a basis for the solution. Through the proposed improved method, which dynamically adjusts control parameters, the problem converges rapidly to a local minimum. The pseudocode for the improved algorithm is as follows, see Algorithm 2.
**Algorithm 2:** Algorithm for updating the parameters of Improved Alternating Direction Method of Multipliers.**Input: S**, **λ**, **X** = 0, **B** = 0, **V** = 0, δ = 1, ε.**Output:** $X\in C^{M\times N}$ 1. **Begin** 2. **While** error > ε 3.    **U** = **B** − **V** 4.    $\hat{X\;}=\;U-\frac{1}{1+\delta }\;F_{a}^{H}F_{a}UF_{r}^{T}\bar{F_{r}}-F_{a}^{H}S\bar{F_{r}}$ 5.    V = V + X−B 6.    $\hat{B}\;(p,q)\;=\;\max(X(p,q)\;+\;V(p,q)-\lambda /\delta ,0)\frac{X(p,q)\;+\;V(p,q)}{{\left|X(p,q)\;+\;V(p,q)\right|}^{,}}\;p\;=\;1,\;\ldots ,\;P,\;q\;=\;1,\;\ldots ,\;Q.$ 7.    $r_{x}\;=\;{\left\|\hat{B}-\hat{\;X}\right\|}_{F}$$,\;r_{d}\;=\;{\left\|\hat{B}-B\right\|}_{F}$ 8.    $\delta \;=\;\delta \;\cdot \;{\mathrm{Iv}}_{\left(1.1,\;1.5\right)}(r_{x}/2r_{d})$ 9.    ${\mathrm{Iv}}_{\left(1.1,\;1.5\right)}(x)\;=\left\{\begin{array}{c} 1.1,\;x\;<\;1.1\;\;\\ x,\;\;1.1\;\leq \;x\;\leq \;1.5\;\\ 1.5,\;x\;>\;1.5\;\; \end{array}\right.$ 10.     $\hat{V}\left(p,\;q\right)\;=\;\frac{\hat{V}\left(p,\;q\right)}{{\mathrm{Iv}}_{\left(1.1,\;1.5\right)}(r_{x}/2r_{d})},\;p\;=\;1,\;2\ldots ,\;P,\;q\;=\;1,\;2,\;\ldots ,\;Q$ 11.     $X\;=\;\hat{X},\;B\;=\;\hat{B},\;V\;=\;\hat{V}$ 12.     $\mathrm{error}\;={\left\|X-\hat{X}\right\|}_{F}/{\left\|X\right\|}_{F}$ 13. **end**

In the current alternating direction method of multipliers, the update variable δ is initialized to a fixed positive value and remains constant throughout all algorithm iterations. The proposed method employs a piecewise function to adaptively adjust δ, facilitating the selection of an appropriate value. At the same time, the algorithm converges rapidly by dynamically adjusting δ at each iteration. The initial value of δ is set to 1. The value of λ is influenced by the noise level; subsequent experiments will discuss the selection of λ.

## 4. Experimental Simulation

### 4.1. Radar and Spacecraft Simulation Models

**Figure 1:**
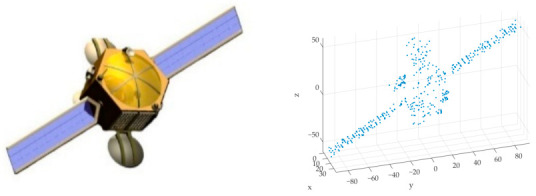
CAD models of spatial targets and scatter point models.

The simulated radar used in the experiment was located at 40.39° N, 116.69° E, at an altitude of 100 m in the geocentric coordinate system. The software used for the experiment was MATLAB R2020b, and the hardware environment primarily consisted of a CPU with 32 GB of RAM. The specific radar parameter information is shown in the table below. The simulated imaging time for the experiment was 28 January 2025, at 13:48:12. The TLE corresponding to the space target’s orbit was used as input for the calculations. The TLE data was sourced from publicly available data on the NASA website. Assuming that the scattering coefficients of the small, abstract scatterers forming the radar echoes in the CAD model simulation are similar—that is, the set of scatterers on the abstract model’s contour—the final imported spatial target CAD model and the generated scatterers are shown in the figure below. This assumption facilitates the simulation process and computations, see Figure 1 and Table 1.

### 4.2. Evaluation Criteria

Image entropy draws on the concept of “information entropy” from statistics. Image entropy is a pure numerical value derived from the probability distribution of each gray level; it is measured in bits and is physically dimensionless. In inverse synthetic aperture radar (ISAR) imaging, the energy values of each pixel are aggregated; each pixel can be regarded as an event, and the corresponding energy value is interpreted as the probability of that event occurring. In general, the better the image focus, the fewer pixels the target occupies, and the lower the corresponding image entropy. Image entropy is defined as

(10) $$E_{x}=-\sum_{i=1}^{M}\sum_{j=1}^{N}\frac{{\left|X(i,\;j)\right|}^{2}}{\mathrm{sumx}}\ln\frac{{\left|X(i,\;j)\right|}^{2}}{\mathrm{sumx}},$$

in which

(11) $$\mathrm{sumx}\;=-\sum_{i=1}^{M}\sum_{j=1}^{N}{\left|X(i,\;j)\right|}^{2},$$

where **X** is the image to be evaluated, with dimensions *M* × *N*.

Image contrast refers to the difference between the brightest and darkest parts of an image; it determines the tonal range and visual impact. High-contrast images appear sharper and have more vivid colors. In ISAR imaging, image contrast reflects the difference in brightness between the target scattering points and the background noise in the image. The higher the contrast, the clearer and more prominent the target contours and structures appear in the image. One of its core metrics is the target-to-background contrast ratio, typically expressed as

(12) $$C_{I\mathrm{SAR}}=\frac{\sigma }{\mu }=\frac{\sqrt{\frac{1}{P}\sum_{M,N}{\left|I(M,\;N)-\mu \right|}^{2}}}{\frac{1}{P}\sum_{M,N}\left|I(M,\;N)\right|},$$

where μ is the average amplitude of the pixels in the target region, and σ is the average amplitude of the background region. The larger this ratio, the more distinct the target is from the background, and the better the image quality is typically.

Normalized Mean Square Error (NMSE) is a dimensionless performance metric widely used in regression modeling and signal processing. It is defined as the ratio of the mean square error (MSE) to the variance of the observed data. Through normalization, this metric effectively eliminates the influence of absolute data units and scale differences, making it possible to compare the predictive accuracy of datasets of different magnitudes or with different physical backgrounds. Numerically, NMSE < 1 indicates that the model’s prediction outperforms the simple mean prediction; It intuitively reflects the proportion of variance explained by the model. It is particularly suitable for estimating communication channels, computational fluid dynamics, and assessing model robustness in multi-source heterogeneous data.

### 4.3. Experimental Results

**Figure 2:**
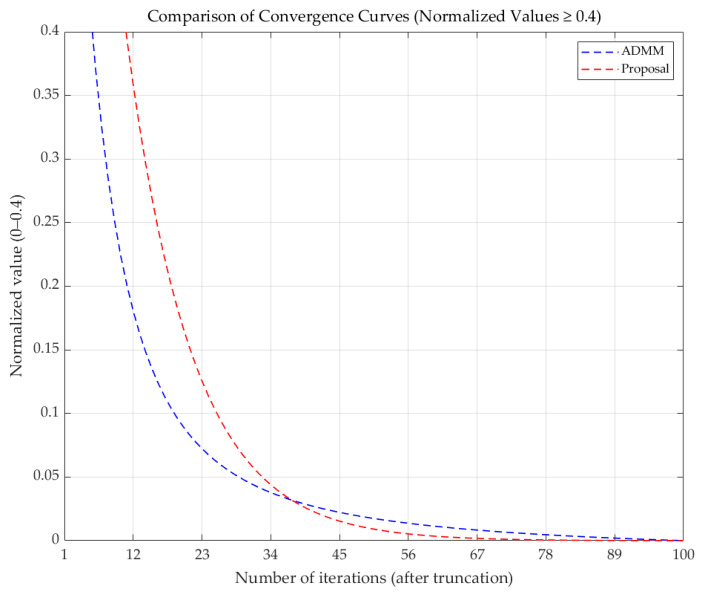
Convergence of the results for the two methods.

The advantages of the improved algorithm described in Chapter 2 are demonstrated by comparing it with the traditional parameter amplitude correction method, OMP (Orthogonal Matching Pursuit), and the unmodified ADMM method. The convergence rates of the improved algorithm are first compared with those of the original direction-of-multipliers method. Under a sparsity of K = 60% and an SNR of −10 dB, the objective function is defined as the relative error, and its behavior as a function of the number of iterations is shown in the Figure 2.

The comparison results show that the improved algorithm converges faster than the original algorithm around the 40th iteration and yields better results. It can be concluded that the improved algorithm converges to a smaller error more quickly than the original algorithm; the improvement begins to outperform the original algorithm around the 38th iteration, and the relative increase in speed is modest.

**Figure 3:**
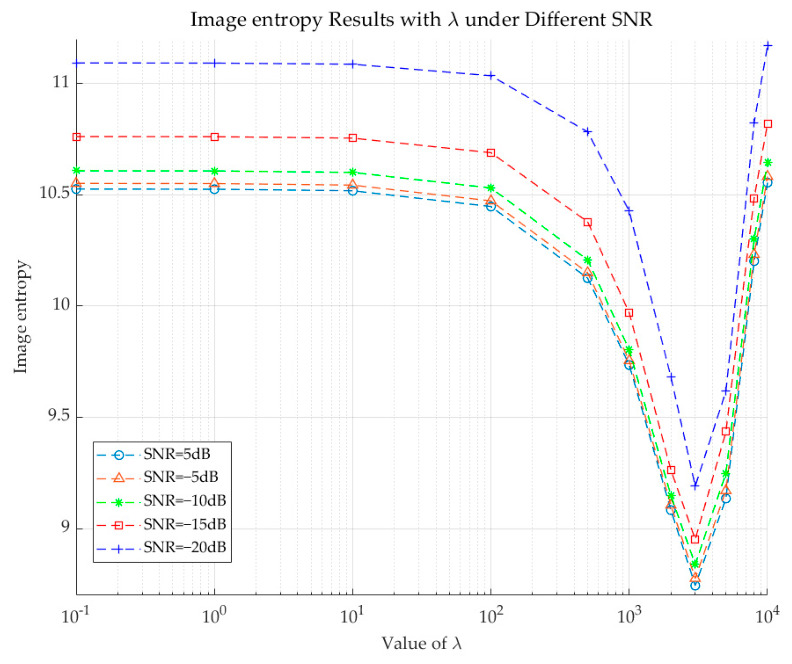
The relationship between image entropy and the value of λ at different SNR levels.

The following analysis examines the effect of the parameter λ on the imaging results. The improved algorithm proposed in this paper dynamically adjusts λ to adapt to the results of each iteration step, thereby accelerating the algorithm’s convergence. A reasonable value of λ must be selected based on experimental conditions. Therefore, an evaluation criterion strongly correlated with noise levels—namely, image entropy—was selected. Under conditions where the echo data is anchored with a sparsity of K = 60% and an SNR of −10 dB, the improved algorithm was used to find the optimal value of λ across four different noise levels. The resulting variation curve is shown in the Figure 3. Analysis of the curve reveals that, for the four noise levels—with the exception of −30 dB, where the noise level is sufficiently high to dominate the echo—a λ value of 3000 is most appropriate. This value yields the lowest image entropy and demonstrates better adaptability to the noise environment compared to other values.

After selecting the parameters, two metrics—image contrast and NMSE—are used to quantitatively analyze the sparse recovery performance. Higher image contrast indicates clearer imaging, where the scattered points are more easily distinguishable from the background. The closer the NMSE is to 0, the better the detail and completeness of the recovered image compared to images obtained under ideal conditions. In the image, the horizontal axis represents the range direction, corresponding to the projected distance of the target’s scatter point along the radar line of sight; the vertical axis represents the cross-range direction, corresponding to the lateral distance perpendicular to the line of sight, obtained by inverting the Doppler shift caused by the target’s rotation around the radar line of sight; together, these axes form a two-dimensional projection of the target’s backscatter intensity in the radar observation coordinate system. After calibration, the units are typically in meters, but this is not, in essence, a true geometric optical image.

**Figure 4:**
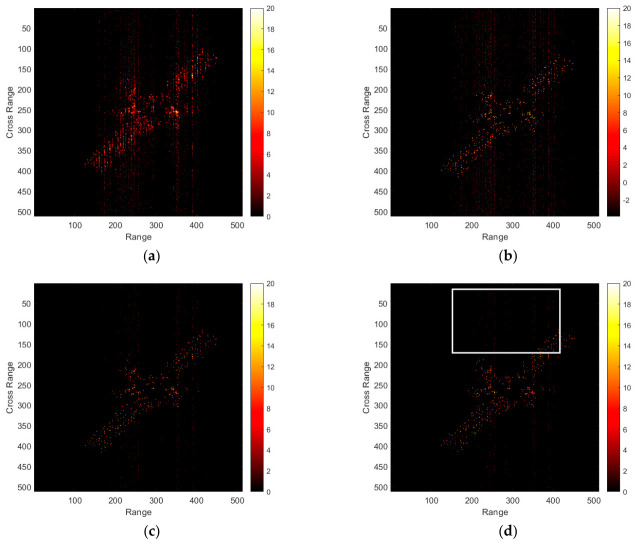
Imaging results for the four methods at 60% sparsity and a noise level of −10 dB: (**a**) Amplitude and Phase Correction. (**b**) OMP. (**c**) ADMM. (**d**) Proposal.

**Figure 5:**
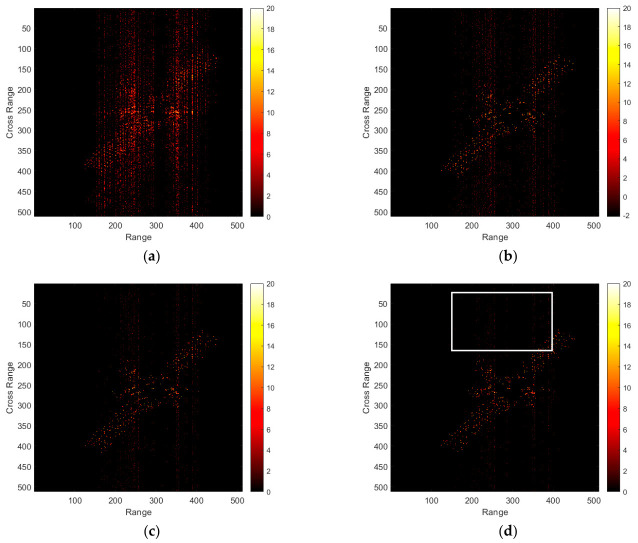
Imaging results for the four methods at 70% sparsity and a noise level of −10 dB: (**a**) Amplitude and Phase Correction. (**b**) OMP. (**c**) ADMM. (**d**) Proposal.

**Figure 6:**
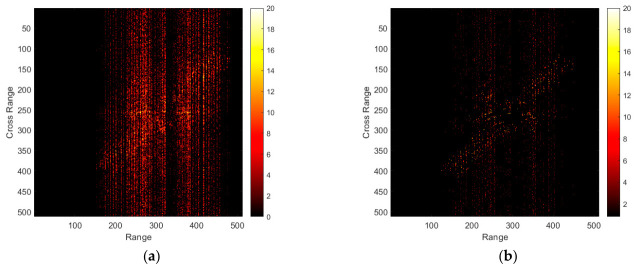

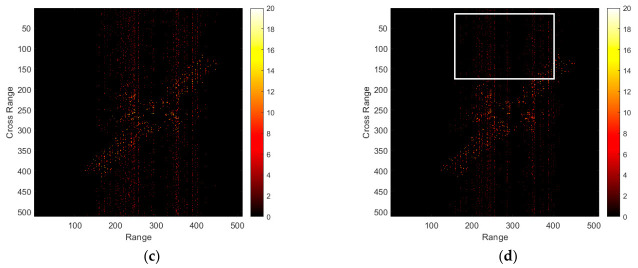
Imaging results for the four methods at 80% sparsity and a noise level of −10 dB: (**a**) Amplitude and Phase Correction (APC). (**b**) OMP. (**c**) ADMM. (**d**) Proposal.

The following table presents the imaging contrast, image entropy, and NMSE values for three sparsity levels (60%, 70%, and 80%) with an anchor noise level of −10 dB. Different levels of sparsity reflect the varying degrees of influence from the undersampling of observations, allowing for a better assessment of the algorithm’s sparsity reconstruction performance, see Table 2 and Figure 4, Figure 5 and Figure 6.

**Figure 7:**
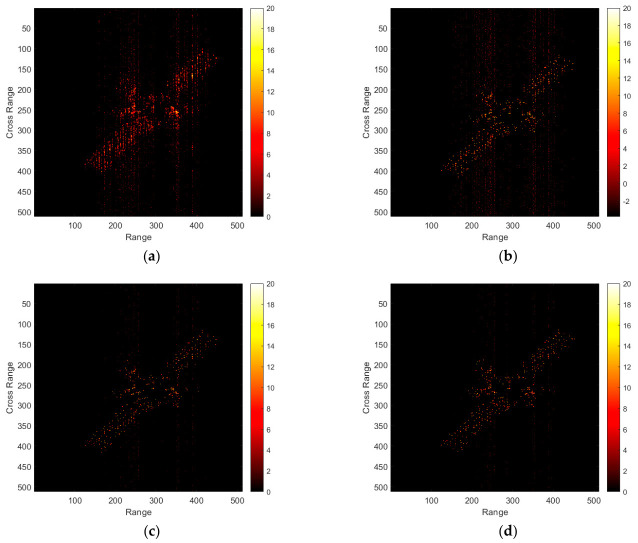
Imaging results for the four methods at 60% sparsity and a noise level of 10 dB: (**a**) Amplitude and Phase Correction. (**b**) OMP. (**c**) ADMM. (**d**) Proposal.

**Figure 8:**
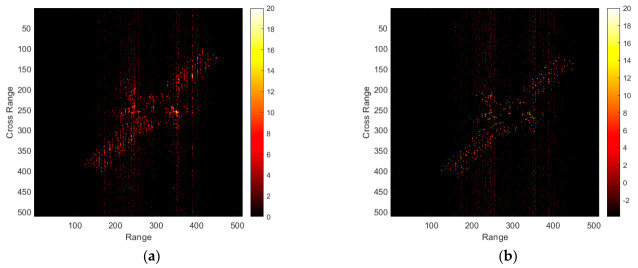

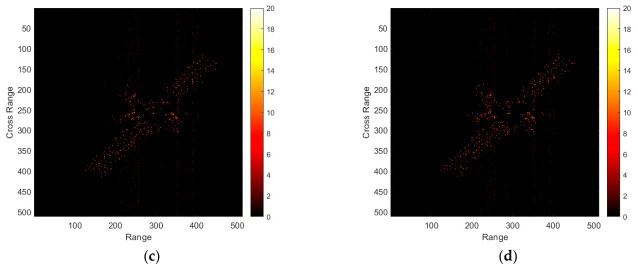
Imaging results for the four methods at 60% sparsity and a noise level of −10 dB: (**a**) Amplitude and Phase Correction. (**b**) OMP. (**c**) ADMM. (**d**) Proposal.

**Figure 9:**
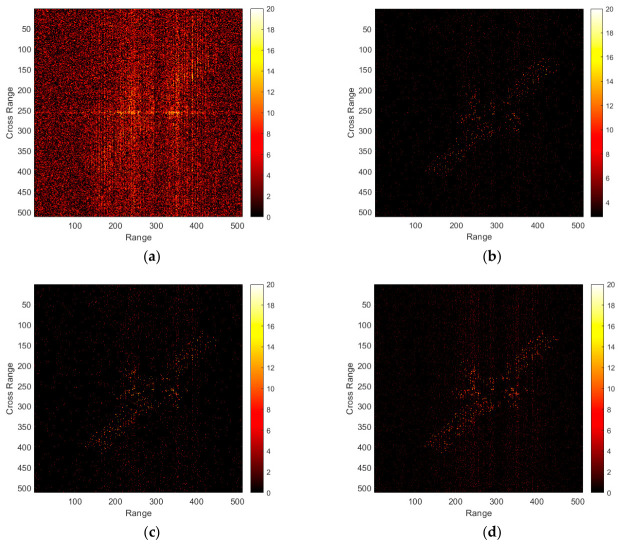
Imaging results for the four methods at 60% sparsity and a noise level of −30 dB: (**a**) Amplitude and Phase Correction (APC). (**b**) OMP. (**c**) ADMM. (**d**) Proposal.

The following table shows the imaging comparisons, entropy values of the images, and NMSE results for an anchoring sparsity of 60% at three noise levels: 10 dB, −10 dB, and −30 dB. In the noise model used in this paper, a significant portion of the noise represents sudden, high-amplitude, short-duration strong pulse interference during the imaging process, such as intentional human interference and sudden cosmic radio frequency pulses. The remaining noise levels are generated through simulations and modeling of real-world spatial environments to better evaluate the performance of the four methods, see Table 3 and Figure 7, Figure 8 and Figure 9.

**Figure 10:**
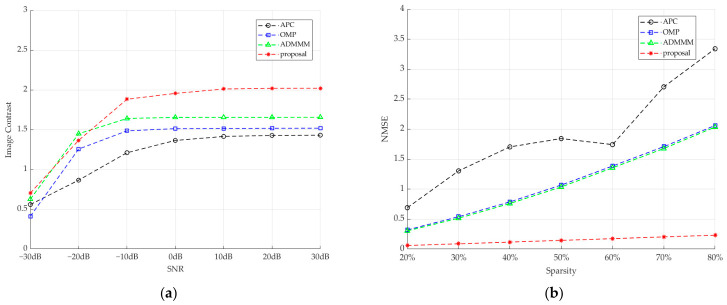
Comparative Analysis of Different Noise Levels and Sparsity Levels: (**a**) variation in image contrast for the four methods at different SNR levels with a sparsity of 60%; (**b**) variation in image contrast for the four methods at a noise level of −10 dB as a function of sparsity level.

The Figure 10 shows a comparative analysis of different noise levels and sparsity levels. The left graph displays the analysis curve for the image contrast evaluation metric under an anchoring sparsity of 60%. Because noise generates artifacts, image contrast more accurately reflects whether the scattering points of the imaged subject have higher energy relative to the noise; this appears in the image as the clarity and prominence of the subject. Under low-noise conditions, the improved algorithm demonstrates significantly better performance than the original method. As noise gradually overwhelms the echo image, there is a brief period where the original method exhibits higher contrast than the improved method; this is likely due to the generation of high-energy noise. However, under general conditions, the improved algorithm demonstrates better noise recovery performance compared to the original method. The graph on the right shows the analysis of the NMSE evaluation metric under a noise anchor of −10 dB. Since sparsity can affect the integrity of the main subject in the generated image, leading to ghosting and ultimately affecting image quality, the NMSE metric—which reflects image integrity—is used for different sparsity levels. As the sparsity increases—that is, as the proportion of missing echoes rises—the ghosting in the image becomes more pronounced. The closer the NMSE is to 0, the higher the degree of recovery of the image relative to the complete echo. Therefore, the proposed improved algorithm more completely restores the target’s scattering point structure, representing a significant improvement over the original ADMM method.

The following analysis examines the average runtime of the OMP, ADMM, and improved ADMM methods, calculating the average runtime for a single run, five runs, and twenty runs, respectively. An analysis of the results shows that, when the initial δ is set to 1, the ADMM method takes more time to complete because it maintains a fixed δ, whereas the proposed improved ADMM method achieves faster computation speeds by dynamically updating the δ, see Table 4.

## 5. Conclusions

An improved method for simulating sparse imaging of spatial targets using TLE data is proposed. Compared to existing techniques, the technical advantage of this study lies in its ability to effectively enhance imaging performance in simulations under conditions of high sparsity and high noise and to generate imaging results of spatial targets using system simulation methods. The innovation of this study lies in the simultaneous introduction of sparsity processing and an appropriate noise model while generating the echo matrix using TLE data, followed by sparse imaging using the proposed improved ADMM method. The imaging results obtained from this method provide a foundation for future research into additional sparse imaging techniques. The error in the SPG4 model’s imaging at each time step results in a velocity error within an acceptable range for rapidly moving spatial targets. The validity of the echo matrix data based on TLE data simulation has been demonstrated, providing reasonable and applicable data support for the validation of subsequent improved ADMM methods. Experimental results also indicate that this improved method yields superior imaging results under adverse conditions compared to traditional imaging methods and demonstrates faster parameter convergence than the ADMM method. However, since the experiments were conducted using simulated data, further validation and analysis using real radar echo data are required. The inverse synthetic aperture radar imaging simulation technique proposed in this paper can effectively process highly sparse and noisy data in practical applications and can simulate the relative attitude of spatial targets with respect to the radar, demonstrating significant practical value.

## Figures and Tables

**Table 1:** Simulated Radar Parameter Settings.

Radar Parameters	Values
Transmit Signal Carrier Frequency (*f*_c_)	5.4 × 10^9^ Hz
Transmit Signal Bandwidth (**B**)	5.12 × 10^8^ Hz
Transmit Pulse Width (**T**)	3 × 10^−5^ s
Pulse Repetition Frequency (PRF)	3500 Hz
Number of Distance Samples (*N*)	512
Number of Accumulated Pulses (*M*)	512

**Table 2:** Experimental results showing image contrast and NMSE values at an SNR of −10 dB.

Sparsity	Evaluation Criteria	APC	OMP	ADMM	Proposal
20%	Image Contrast	1.6836	3.5186	3.6783	**3.7309**
	NMSE	0.81494	0.32628	0.30778	**0.02105**
30%	Image Contrast	1.5638	2.9484	3.1189	**3.2922**
	NMSE	1.2988	0.54568	0.51946	**0.031467**
40%	Image Contrast	1.3951	2.4076	2.5668	**2.9864**
	NMSE	1.51	0.79125	0.76353	**0.040998**
50%	Image Contrast	1.2793	1.9546	2.1271	**2.6494**
	NMSE	1.8531	1.073	1.0407	**0.051257**
60%	Image Contrast	1.1915	1.4123	1.5567	**1.8535**
	NMSE	1.8797	1.3885	1.3584	**0.061278**
70%	Image Contrast	1.1272	1.0704	1.2018	**1.7402**
	NMSE	2.7673	1.7119	1.6808	**0.07122**
80%	Image Contrast	1.0364	0.66519	0.76442	**1.6289**
	NMSE	3.271	2.0497	2.0221	**0.081227**

**Table 3:** Experimental results showing image contrast and NMSE values at 60% sparsity.

SNR	Evaluation Criteria	APC	OMP	ADMM	Proposal
30 dB	Image Contrast	1.4307	1.5183	1.6566	**2.0193**
	NMSE	1.7046	0.7871	0.76004	**0.041219**
20 dB	Image Contrast	1.4276	1.5174	1.6563	**2.0184**
	NMSE	1.7061	0.7871	0.76036	**0.041219**
10 dB	Image Contrast	1.4138	1.5153	1.6545	**2.0103**
	NMSE	1.7046	0.78757	0.76037	**0.041221**
0 dB	Image Contrast	1.3643	1.5125	1.6539	**1.9551**
	NMSE	1.7094	0.78787	0.76075	**0.041234**
−10 dB	Image Contrast	1.1915	1.4123	1.5567	**1.8535**
	NMSE	1.51	0.79125	0.76353	**0.0419198**
−20 dB	Image Contrast	0.86497	1.2557	1.4487	**1.4656**
	NMSE	2.159	0.85183	0.80451	**0.041968**
−30 dB	Image Contrast	0.55835	0.41135	0.62583	**0.70292**
	NMSE	6.0541	1.4956	1.2761	**0.046494**

**Table 4:** Number of experiments and average experiment runtime for each method.

Methods	OMP	ADMM	Proposal
1	7.7224 s	78.2378 s	5.6104 s
5	7.7794 s	79.6012 s	5.8222 s
20	7.8039 s	79.4511 s	5.8671 s

## Data Availability

The data presented in this study are available on request from the corresponding author. The data are not publicly available due to the request of the funder.

## Abbreviations

The following abbreviations are used in this manuscript:

ISAR	Inverse synthetic aperture radar
TLE	Two-line element
SNR	Signal-to-noise ratio
APC	Amplitude and Phase Correction
OMP	Orthogonal Matching Pursuit
ADMM	Alternating Direction Method of Multipliers
NMSE	Normalized Mean Square Error
